# Dynamic multi-pinhole collimated brain SPECT of Parkinson’s disease by [^123^I]FP-CIT: a feasibility study of fSPECT

**DOI:** 10.1038/s41598-024-57152-5

**Published:** 2024-03-19

**Authors:** Filip L. H. Fredensborg, Kasper Thilsing-Hansen, Jane A. Simonsen, Peter Grupe, Ziba A. Farahani, Christian W. Andersen, Albert Gjedde, Svend Hvidsten

**Affiliations:** 1 Department of Nuclear Medicine, Odense University Hospital, Odense, Denmark; 2 Department of Clinical Research, University of Southern Denmark, Odense, Denmark; 3 Translational Neuropsychiatry Unit, Aarhus University and Aarhus University Hospital, Aarhus, Denmark; 4 Department of Neuroscience, University of Copenhagen, Copenhagen, Denmark; 5 Department of Neurology and Neurosurgery, McGill University, Montréal, Québec Canada

**Keywords:** Parkinson's disease, Transporters in the nervous system, Molecular imaging

## Abstract

We investigated the feasibility of using a dopamine transporter (DaT) tracer ligand ([^123^I]FP-CIT) along with novel multi-pinhole brain collimators for dynamic brain single photon emission computed tomography (SPECT) in suspected Parkinson's disease patients. Thirteen patients underwent dynamic tracer acquisitions before standard imaging. Uptake values were corrected for partial volume effects. Specific binding ratio (SBR_calc_) was calculated, reflecting binding potential relative to non-displaceable binding (BP_ND_) in the cortex. Additional pharmacokinetic parameters (BP_ND_, R_1_, k_2_) were estimated using the simplified reference tissue model, revealing differences between Kahraman low-score (LS) and high-score (HS) groups. Results showed increasing striatal tracer uptake until 100 min post-injection, with consistent values afterward. Uptake and SBR_calc_ ratios matched visual assessment. LS patients had lower putamen than caudate nucleus tracer uptake, decreased BP_ND_ values, while R_1_ and k_2_ values were comparable to HS patients. In conclusion, dynamic multi-pinhole SPECT using DaT tracer with the extraction of pharmacokinetic parameters is feasible and could help enable early differentiation of reduced and normal DaT values.

## Introduction

Neurological disorders are among the world’s leading causes of disability; among these, Parkinson’s disease (PD) is the fastest-growing neurodegenerative disease with a prevalence of more than 6 million afflicted individuals worldwide, projected to more than double in coming decades^1,2^. PD is characterized by loss of dopamine transporter (DaT) sites in striatum, with cardinal motor symptoms of bradykinesia, tremor, rigidity, and postural instability. Besides these signs, however, a wide spectrum of manifestations include non-motor symptoms (e.g., constipation, dysphagia, sleeping disorder, depression). The sole treatment consists of symptomatic interventions intended to raise dopamine availability. The most common medication is the amino acid L-dihydroxyphenylalanine (L-DOPA) that transports across the blood–brain barrier and undergoes conversion to dopamine in neurons equipped with the appropriate enzyme^3,4^.

PD primarily is a clinical diagnosis, but so-called functional single-photon emission computed tomography (fSPECT) can aid the diagnosis with the ^123^I-labeled cocaine analogue ioflupane (N-3-fluoropropryl-2β-carbomethoxy-3β-(4-iodophenyl)nortropane ([^123^I]FP-CIT) that binds with high affinity to presynaptic DaT proteins^5^. The imaging reveals neurodegeneration by loss of dopaminergic neurons in the nigrostriatal pathways as indication of PD^3,5^. The EANM/SNMMI guidelines for dopaminergic imaging of parkinsonian syndromes state that DaT SPECT uses a gamma camera equipped with parallel-hole collimators at 3–6 h post-injection (p.i.)^5^. The evaluation is based mainly on visual interpretation although semiquantitative measures often are used to aid the assessment.

With the recent introduction of multi-pinhole collimators into the clinical setting, large gains of sensitivity and resolution significantly may improve overall image quality and visual interpretation^6^. Initial tests with a three-headed SPECT/CT camera (AnyScan Trio SC, Mediso Medical Imaging System, Hungary) with multi-pinhole collimators, designed specifically for brain imaging, reveal a 3.9-fold increase of peak spatial sensitivity (700 cps/MBq) and a spatial resolution of approximately 5 mm half that of conventional two-headed SPECT with parallel-hole collimators^7,8^. The improved resolution lowers partial volume effects (PVE) and improves delineation of striatum while the higher sensitivity enables dynamic scanning and time framing with the potential to more accurately reveal tracer dynamics and hence the pharmacokinetics of [^123^I]FP-CIT.

We designed the present study to explore the clinical feasibility of dynamic multi-pinhole fSPECT to investigate pharmacokinetic modelling of [^123^I]FP-CIT in a series of patients suspected of PD, compared to conventional clinical imaging. We completed the study also as an exploration of the technical abilities of the multi-pinhole system. A primary objective was thus to examine the feasibility of dynamic DaT SPECT (fSPECT). Additional objectives included the acquisition of dynamic and static measurements that would aid the diagnostic process, and the study of tracer dynamics with the goal of optimizing the conventional imaging protocol.

## Materials and methods

### Subjects

Thirteen patients (three women) referred to the department for imaging by DaT SPECT underwent acquisitions of dynamic brain scans of [^123^I]FP-CIT uptake by means of fSPECT. The patients gave informed consent prior to participation.

### Procedures and image acquisition

We imaged patients with the three-headed Mediso AnyScan Trio SC SPECT/CT device equipped with three multi-pinhole collimators. Following low-dose CT (120 kV, 20 mAs, 1 s rotation, 20 mm collimation, 1.5 pitch), we intravenously injected the patients with 120 MBq [^123^I]FP-CIT at the time of initiation of the dynamic fSPECT protocol. The protocol included the following steps: At 0–20 min, we completed four 5-min SPECT acquisitions (45 projections, 20 s/projection), immediately followed by repeated 10-min SPECT acquisitions (60 projections, 30 s/projection) as frequently as accepted by the patient. After a break, we planned to achieve at least two 10-min SPECT acquisitions beginning at about two hours post-injection (p.i.) to avoid large time gaps in the data sets. After the break, we redid the low-dose CT scan before continuing the SPECT protocol. At three hours p.i., we initiated the imaging of the patient according to standard clinical SPECT procedures (15 min, 90 projections, 30 s/projection). After the first five patients, we increased the number of projections. The dynamic protocol then consisted of 4 × 6-min fSPECT (60 projections, 18 s/projection) followed by repeated 12-min fSPECT acquisitions (72 projections, 30 s/projection) for as long as tolerated by the patient, or till the end of the planned protocol.

### Post-processing

All image acquisitions underwent reconstruction utilizing Interview XP 3 Workstation (Mediso Medical Imaging System) with the Tera-Tomo Q reconstruction framework developed by Mediso. This framework utilizes a regularized ordered subset expectation maximization (OSEM) reconstruction method, which integrates a comprehensive model of the entire system geometry and accounts for the physical interactions, including scatter and attenuation, of gamma photons through Monte Carlo simulation. Transaxial images were reconstructed with regularization filter configured to adaptive bilateral high^9^, with 54 iterations with 3 subsets, while transaxial image dimension were defined to 108 × 108 pixels, Monte Carlo quality was set to medium. The resulting transaxial images had an isotropic voxel size of 2.13 mm. The acquired field of view measured 19 cm in the axial direction and 23 cm in the transaxial direction. All reconstructions underwent quantitative analysis, providing decay-corrected values in Becquerels per milliliter (Bq/ml), facilitating comparison of tracer uptake across various image acquisitions within and among patients.

### Image analysis

Senior nuclear physicians (PG and ZA) interpreted the 3-h p.i. image, independently, with any differences resolved by discussion. Interpretation rested on the specific binding ratio calculated by the commercially available DaTQUANT™ program (GE Healthcare, Brondby, Denmark), SBR_DaTQUANT_, as mainly based on the visual grading system of Kahraman et al.^10^ with grade 5 indicating normal tracer uptake and lower grades indicating lower tracer uptake in striatum. For the analyses, we divided the patients into two groups, based on the 3-h p.i. interpretations as low-score (LS, grades 1–3) and high-score (HS, grades 4–5) groups.

**Figure 1:**
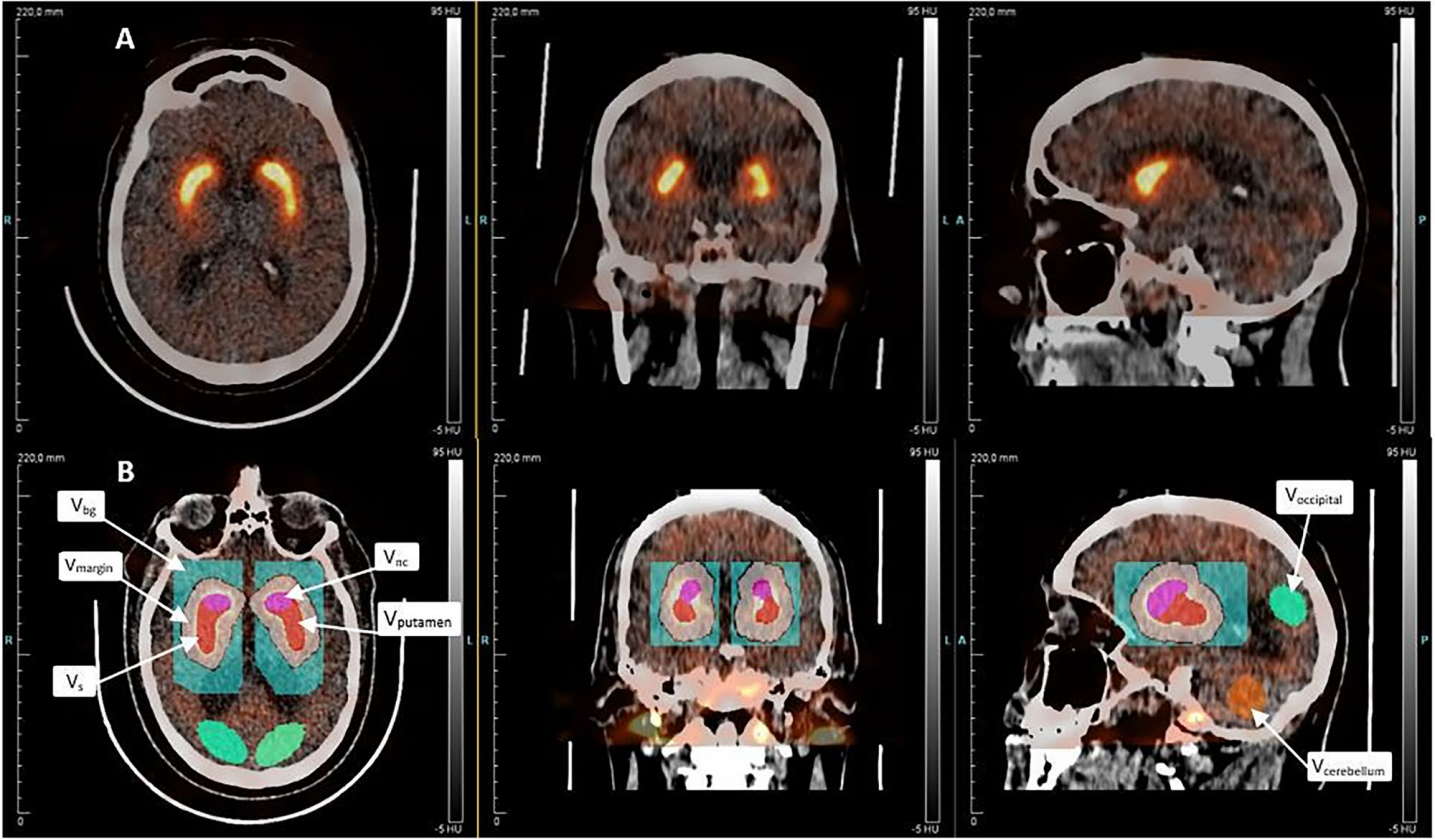
Volumes of interest as shown on a high-score patient scan. Pink: V_caudate nucleus_. Red: V_putamen_. V_S_ = V_caudate nucleus_ + V_putamen_. White: V_margin_. Blue (rectangularly shaped): V_BG_. Green: Occipital cortex. Orange: Cerebellum.

Using Inveon Research Workplace 4.2 (Siemens Molecular Solutions, Knoxville, Tennessee, USA), we adjusted for movement between frames by manual co-registration of the different SPECT images. We assumed a standard left and right striatal volume of 8.7 ml on either side, based on healthy controls reported previously^11,12^. As illustrated in Fig. 1, we created several volumes (V) of interest, beginning with a right-sided standard volume of striatum, V_S_. The same volumes were applied to all patients. We manually aligned the standard striatal volume V_S_ to encompass the maximum activity concentration of striatum. We created a new volume around V_S_ by adding a 10 mm wide margin around V_S_ in all directions (V_margin_), intended to include spill-over from PVE and possible minor movement artefacts and would lower the impact of inter-individual differences in striatal volume. V_margin_ did not include V_S_. To correct the V_margin_ for activity from spill-in from the surrounding tissue, we created a rectangular-shaped background volume (BG) around V_margin_. We divided the V_S_ into anteromedial and posterolateral volumes to delineate the caudate nucleus and putamen, respectively. We drew all right-sided volumes on images from the first patient, mirrored for the contralateral hemisphere and used as template for all patients going forward, and adjusted the VOIs placement and orientation individually, without changing the size or shape. In addition, we drew reference volumes in cerebellum and occipital cortex, taking care not to include venous sinuses or skull. Using an modified version of the partial correction technique of Tissici-Bolt^13^ the mean activity concentration of striatum (C_S_) was adjusted for spill-over effects by means of the relationship,

1 $$C_{{S\left( {adj.} \right) }} = C_{S} + \frac{{V_{margin} }}{{V_{S} }}\left( {C_{margin} - C_{BG} } \right)$$

We adjusted the activity in putamen and caudate nucleus by using Eq. (1) with only half of volume V_margin,_ as the volumes of putamen and caudate nucleus each constituted approx. 50% of the striatal volume.

We calculated the specific binding ratio (SBR_calc_) with mean activity concentrations from Eq. (1) and occipital cortex as the reference,

2 $$SBR_{calc} = \frac{{C_{{S\left( {adj.} \right)}} - C_{occipital} }}{{C_{occipital} }}$$

We also extracted SBR_DaTQUANT_ values from 3-h images as done in the clinical setting.

The method was initially validated utilizing an anthropomorphic basal ganglia phantom provided by RSD (Radiology Support Devices Inc., Long Beach, CA, USA). The discrete volumes corresponding to the striatal and brain regions of the phantom were filled and imaged twice with varying ^123^I activity concentrations, resulting in true specific binding ratio (SBR) values of 1.0, 2.5, 3.9, and 9.2. Employing Eqs. (1) and (2), the observed SBR values were determined as 0.9, 2.3, 3.8, and 8.0. The accuracy of the measured brain volume activity concentration (Bq/ml) was confirmed to be within 5% in both imaging sessions.

### Dynamic parameters

The binding potential of bound ligand relative to non-displaceable reference activity (*BP*_ND_) is the ratio, at equilibrium, between the concentrations of receptor-bound and non-displaceable (non-specifically bound and free radioligand)^14^ in a specific region. We calculated the value of *BP*_ND_ by means of the simplified reference tissue model (SRTM)^15^ with the occipital cortex as the specified reference region. We also used the SRTM to calculate the relative index of tissue perfusion (*R*_1_) as the ratio between the tracer clearance from plasma to tissue (*K*_1_) for the striatum and the corresponding rate constant for the occipital cortex (*K*’_1_). The third SRTM parameter, *k*_2,_ represents the rate constant of tracer removal from tissue to plasma. We estimated the SRTM parameters (*R*_1,_ *k*_2_, and *BP*_ND_) from the measured data with least-square method *lmfit* implemented in Python with weighted data points, based on the duration of image acquisitions.

Using the model, we obtained estimates of the variables *BP*_ND_, *R*_1_, and *k*_2_ for striatum, as well as for putamen and caudate nucleus separately, of left and right hemispheres, respectively.

### Statistical analyses

We reported all continuous variables as median (min;max) unless otherwise stated. We reported categorical variables by frequencies (percentages). We employed spaghetti plots for visualization purposes per patient, and we applied exploratory linear regression when deemed appropriate. For the regressions of the SRTM model and analyses of pharmacokinetic parameters, χ^2^ values, and standard errors, we used the *lmfit* module in Python. We analyzed SBR_calc_ and completed all graphical presentations with Microsoft EXCEL 2016. We calculated ratios between striatum of both hemispheres by dividing the lowest left or right value by the highest value of the left and right striatum.

### Ethics approval and consent to participate

The study protocol received approval from the institutional review board (Approval Code: 21/39019), but as the study did not entail intervention or storing of tissue, approval from the Danish ethics committee was waived. Approval from local ethics committee was not necessary as the study qualifies as a quality assurance study^16^. All procedures and methods were conducted in strict adherence to applicable guidelines and regulations. Overall, the study was carried out in full compliance with the principles outlined in the Helsinki Declaration. Informed consent was obtained from all individual participants included in the study.

## Results

### Subjects

We tested all patients for the presence of PD. Patient median age was 73 years ranging from 55 to 83 years. We categorized four patients as LS; one patient as grade 2 and three patients as grade 3. Nine patients were catergorized as HS; two patients as grade 4 and seven patients as grade 5. Ten patients (77%) had tremor-dominant symptoms with an equal distribution of left and right lateralization, all LS categorized patients had tremor-dominant symptoms. Three patients (23%) had other primary symptoms, including sleep behavior disorder and motor symptoms besides tremors, or a familial disposition of first degree. Based on referral information, at least two patients had verified vascular comorbidity, including one patient with MRI-verified lacunar infarcts in the caudate nucleus, and one patient suffered from sequelae from a prior hemorrhage outside striatum.

### Dynamic acquisitions

We present original mean activity concentrations of striatum obtained at hourly intervals in Table 1, per patient as well as for caudate nucleus and putamen. The activities appeared to differ between LS and HS patients at 2 h p.i. and tended to do so as early as at 1 h p.i. At all times, the difference between LS and HS patients was more marked in the putamen than in the caudate nucleus (Table 1).

**Table 1:** Adjusted mean activity concentration in hourly intervals, shown by median (min;max).

Time p.i	1 h (N = 13)	2 h (N = 8)^a^	3 h (N = 13)
Striatum			
All patients	15.9 (9.3; 30.7)	14.1 (9.1; 35.4)	19.1 (7.8; 33.6)
LS	12.6 (9.5; 14.3)	12.4 (9.1; 15.3)	11.8 (7.8; 15.5)
HS	17.7 (9.3; 30.7)	19.6 (12.7; 35.4)	19.7 (12.2; 33.6)
Putamen			
All patients	15.6 (9.1; 32.0)	13.4 (7.9; 35.5)	18.4 (6.4; 32.9)
LS	12.0 (9.1; 13.6)	10.7 (7.9; 13.7)	9.9 (6.4; 14.2)
HS	17.3 (10.0; 32.0)	19.2 (12.9; 35.5)	19.6 (12.0; 32.9)
Caudate nucleus			
All patients	15.4 (8.6; 30.0)	14.4 (10.3; 35.7)	19.4 (9.2; 34.7)
LS	13.2 (9.9; 14.6)	13.9 (10.3; 16.5)	13.8 (9.2; 16.3)
HS	17.6 (8.6; 30.0)	19.2 (12.6; 35.7)	20.0 (12.4; 34,6)

Mean activity concentration for patients at 1 h, 2 h, and 3 h p.i.

*LS* low score patients, *HS* High score patients.

^a^Four LS patients and four HS patients had an image acquisition at around 2 h p.i.

**Figure 2:**
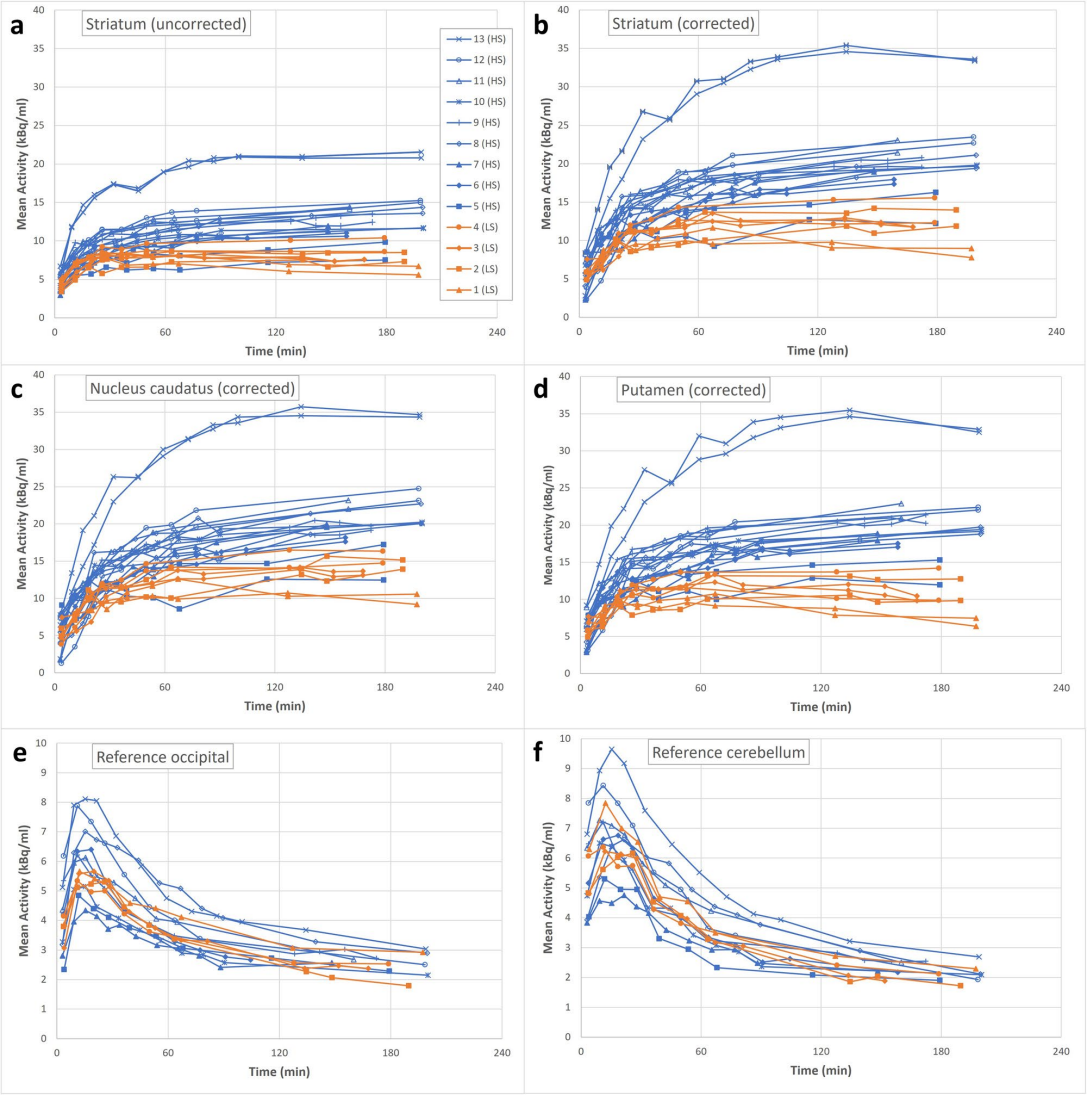
Relation between activity and time. (**a**) Uncorrected mean activity concentration for striatum. (**b**) Striatal mean activity concentration corrected for partial volume and minor movement artefacts. (**c**) Corrected mean activity concentration in the caudate nucleus. (**d**) Corrected mean activity concentration in the putamen. (**e**) Mean activity concentration in the occipital cortex. (**f**) Mean activity concentration in the cerebellum. Each different marker represents a patient with values from both the left and right striatum shown in (**a**–**d**). Patients #1–4 had Karahman scores of < 4 at 3-h images and are illustrated in orange.

We used data from the dynamic SPECT images to construct the time-activity curves (TAC) presented in Fig. 2. Figure 2a shows uptake in V_s_, while Fig. 2b–d show corrected uptake values according to Eq. (1). Uncorrected uptake values were approximately 30% lower than adjusted values; however, the degree of underestimation was smaller for patients with lower uptake values (e.g., patient #1). Figure 2a–d show two TAC per patient representing both left and right striatum. The shapes of TACs in Fig. 2a–d were similar for patients with an initial increase prior to 100 min, followed by a plateau. From Fig. 2a–d, TACs from HS and LS patients diverted after about an hour and onwards, although patient #5 appeared to maintain low uptake consistent with the LS patients, most clearly seen in the case of putamen (Fig. 2d). We note that one patient (#13) appeared to have higher uptake values mostly in striatum (Fig. 2a,b), albeit also in reference tissues (Fig. 2e,f). Regarding reference volumes, the occipital cortex (Fig. 2e) presented with the lower mean activity concentration initially, compared to cerebellum (Fig. 2f).

**Figure 3:**
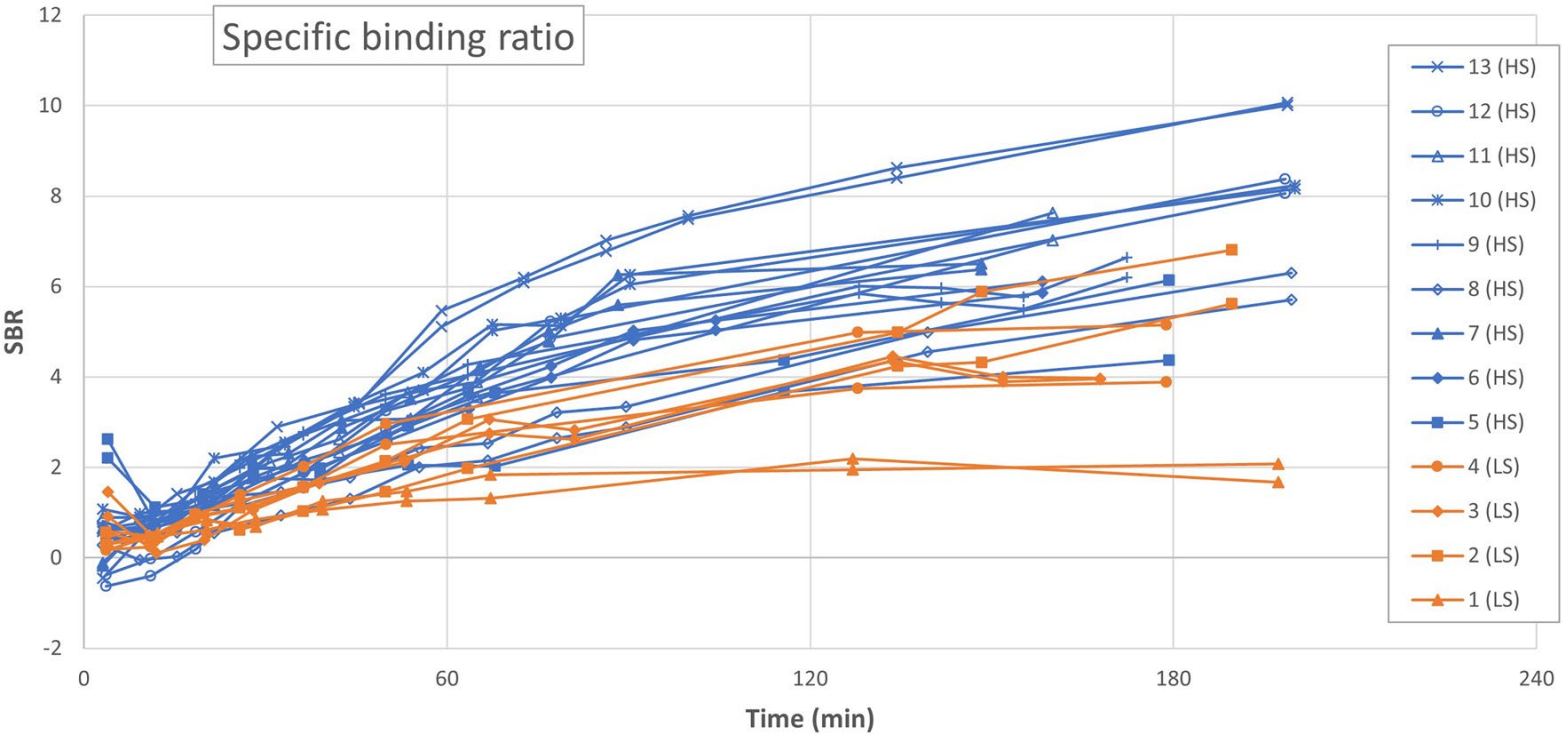
Calculated specific binding ratio (SBR_calc_) for all patients throughout the scan duration.Each patient is represented by a different mark and illustrated with a dataset from both the left and the right striatum. Patients with #1–4 had Karahman scores of < 4 at 3 h p.i. images and are illustrated in orange.

The striatal SBR_calc_ values (Fig. 3) had a more linear course than TACs. The majority of patients presented with ratios of 4–8 at 3 h p.i. As expected, we note a correlation between SBR_calc_ values (Fig. 3) and mean activity concentrations in the striatum (Fig. 2a–d), because the majority of patients with low SBR_calc_ had low striatal activity concentrations, while the patients with the higher SBR_calc_ values had higher striatal activity concentrations at all times of the imaging. We present the values of 3 h p.i. in Table 2. In agreement with the difference between uncorrected and adjusted uptake values, SBR_DaTQUANT_ values reached roughly 2/3 of the values of SBR_calc_. Table 3 presents averages of the 3-h SBR_calc_ values for each patient group. HS patients presented with putamen-caudate ratios of close to unity, while the LS group members presented with distinctly lower values of SBR_calc_ in putamen than in caudate nucleus (Table 3).

**Table 2:** Static and dynamic parameters related to the left and right striatum for each patient.

Pt. #	Grade	Age (y)	Sex (M/F)	Tremor side	Mean activity 3 h p.i. (kBq/ml)		SBR				Model parameters by SRTM					
							Calculated		By DaTQUANT		BP_ND_		R_1_		K_2_	
					Right	Left	Right	Left	Right	Left	Right	Left	Right	Left	Right	Left
1	2	76	M	L	7.8	9.0	1.67	2.07	1.15	1.56	1.82	1.86	1.25	1.19	0.046	0.069
2	3	59	M	R	14.0	11.8	6.80	5.61	3.08	2.69	6.55	6.85^a^	1.43	1.24	0.042	0.028
3	3	74	M	R	11.8	11.8	3.97	3.95	2.47	2.38	3.88	3.76	1.10	1.23	0.054	0.058
4	3	77	M	R	15.5	12.3	5.15	3.88	4.04	3.23	5.14	3.66	1.38	1.35	0.064	0.061
Median (min;max)		75 (59;77)			12.9 (7.8;15.5)	11.8 (9.0;12.3)	4.56 (1.67;6.80)	3.92 (2.07;5.61)	2.78 (1.15;4.40)	2.54 (1.56;3.23)	4.51 (1.82;6.55)	3.71 (1.86;6.85)	1.32 (1.10;1.43)	1.24 (1.19;1.35)	0.050 (0.042;0.064)	0.060 (0.028;0.069)
Combined median (min;max)					11.8 (7.8;15.5)		3.96 (1.67;6.80)		2.58 (1.15;4.04)		3.82 (1.82;6.85)		1.25 (1.10;1.43)		0.056 (0.028;0.069)	
5	4	73	M	L	16.3	12.2	6.13	4.36	4.54	3.23	6.68	7.20^a^	1.98	1.98	0.049	0.027
6	4	78	F	R	17.3	18.0	5.86	6.11	3.82	3.81	7.52	7.17	1.29	1.25	0.050	0.056
7	5	55	M	N/A	19.2	18.9	6.50	6.38	4.80	5.03	8.04	7.67	1.08	1.30	0.083	0.079
8	5	56	M	L	21.1	19.4	6.30	5.70	4.39	4.21	9.83	7.79	1.80	1.24	0.030	0.032
9	5	70	F	L	20.8	19.6	6.64	6.20	4.40	4.04	6.75	5.86	1.72	1.34	0.069	0.085
10	5	73	M	L	19.8	19.7	8.23	8.16	4.73	4.83	8.19	7.75	1.72	1.39	0.074	0.084
11	5	83	M	N/A	23.1	21.4	7.63	7.02	3.89	4.06	9.35	8.62	1.89	1.66	0.052	0.049
12	5	69	M	R	22.7	23.5	8.07	8.47	5.60	5.66	7.59	7.81	0.59	0.77	0.060	0.064
13	5	57	F	N/A	33.4	33.6	10.00	10.07	6.50	6.79	9.13	9.00	1.19	0.69	0.096	0.096
Median (min;max)		70 (55;83)			20.8 (16.3;33.4)	19.6 (12.2;33.6)	6.64 (5.86;10.00)	6.38 (4.36;10.07)	4.54 (3.82;6.50)	4.21 (3.23;6.79)	8.04 (6.68;9.83)	7.75 (7.17;9.00)	1.72 (0.59;1.98)	1.30 (0.77;1.98)	0.060 (0.030;0.096)	0.064 (0.027;0.096)
Combined median (min;max)					19.8 (12.2;33.6)		6.57 (4.36;10.07)		4.47 (3.23;6.79)		7.77 (6.68;9.83)		1.32 (0.59;1.98)		0.062 (0.027;0.096)	
Median, all (min;max)		73 (55;83)			19.2 (7.8;33.4)	18.9 (9.0;33.6)	6.50 (1.67;10.00)	6.11 (2.07;10.07)	4.39 (1.15;6.50)	4.04 (1.56;6.79)	7.52 (1.82;9.83)	7.20 (1.86;9.00)	1.38 (0.59;1.98)	1.25 (0.77;1.98)	0.054 (0.030:0.096)	0.061 (0.027;0.096)

The table shows data for all the patients with their grade (according to Kahraman), sex (male/female), tremor side (right-sided or left-sided) and the values, for left and right striatum, of 3 h post-injection (p.i.) mean activity concentrations, specific binding ratio (SBR) (as calculated and as extracted from DaTQUANT), non-displaceable binding potential (BP), R_1_ and K_2_. Patients #1–4 were categorized as low-score (LS) and patients #5–13 were categorized as high-score (HS). Medians (min;max) are presented for data from both right and left striatum and their combined right + left data, for both LS and HS patients. A median (min;max) of all patients’ data from both the left and the right striatum is also presented.

*N/A* Not applicable.

^a^Had standard error rates more than 100%.

**Table 3:** Ratios between striatum as well as caudate nucleus and putamen, shown by median (min;max).

	Ratios between striatum	Putamen/caudate nucleus	
		Left	Right
LS			
SBR_calc_	0.82 (0.75; 1.00)	0.65 (0.53; 0.73)	0.81 (0.45; 0.95)
BP_ND_	0.98 (0.71; 1.03)	0.62 (0.55; 0.65)	0.73 (0.58; 0.82)
R_1_	0.94 (0.89; 1.02)	0.96 (0.84; 1.02)	1.25 (0.85; 1.43)
HS			
SBR_calc_	0.96 (0.71; 0.99)	0.94 (0.65; 0.73)	0.81 (0.45; 0.95)
BP_ND_	0.95 (0.79; 0.99)	0.93 (0.58; 1.19)	0.73 (0.58; 0.82)
R_1_	0.79 (0.58; 0.97)	1.09 (0.81; 1.35)	1.22 (0.85; 1.43)

Ratios between striatum is computed from the lowest value divided by the highest value of left and right striatum; thus 1.00 indicating complete symmetry and lesser/higher values indicating increasing asymmetry. Putamen/caudate nucleus ratio for both left and right striatum. Specific binding ratio (SBR_calc_) calculated from the corrected data of the 3 h p.i.images.

*BP*_*ND*_ non-displaceable binding potential extracted from SRTM, *LS* low score patients, *HS* High score patients.

### SRTM parameters

The regression results of SRTM were consistent with the measured time activity curves. The dynamic parameters extracted from the model had standard errors of less than 2% of the median, except for the *BP*_ND_ of the caudate nuclei of two patients with standard errors of more than 100%. One patient (#5) had lacunar infarcts in the left caudate nucleus with a standard error for the left striatum of 5%. Another patient (#2) had no reported comorbidity. We excluded both patients’ data from the *BP*_ND_, *R*_1_, and *k*_2_ analyses of the left caudate nucleus.

**Figure 4:**
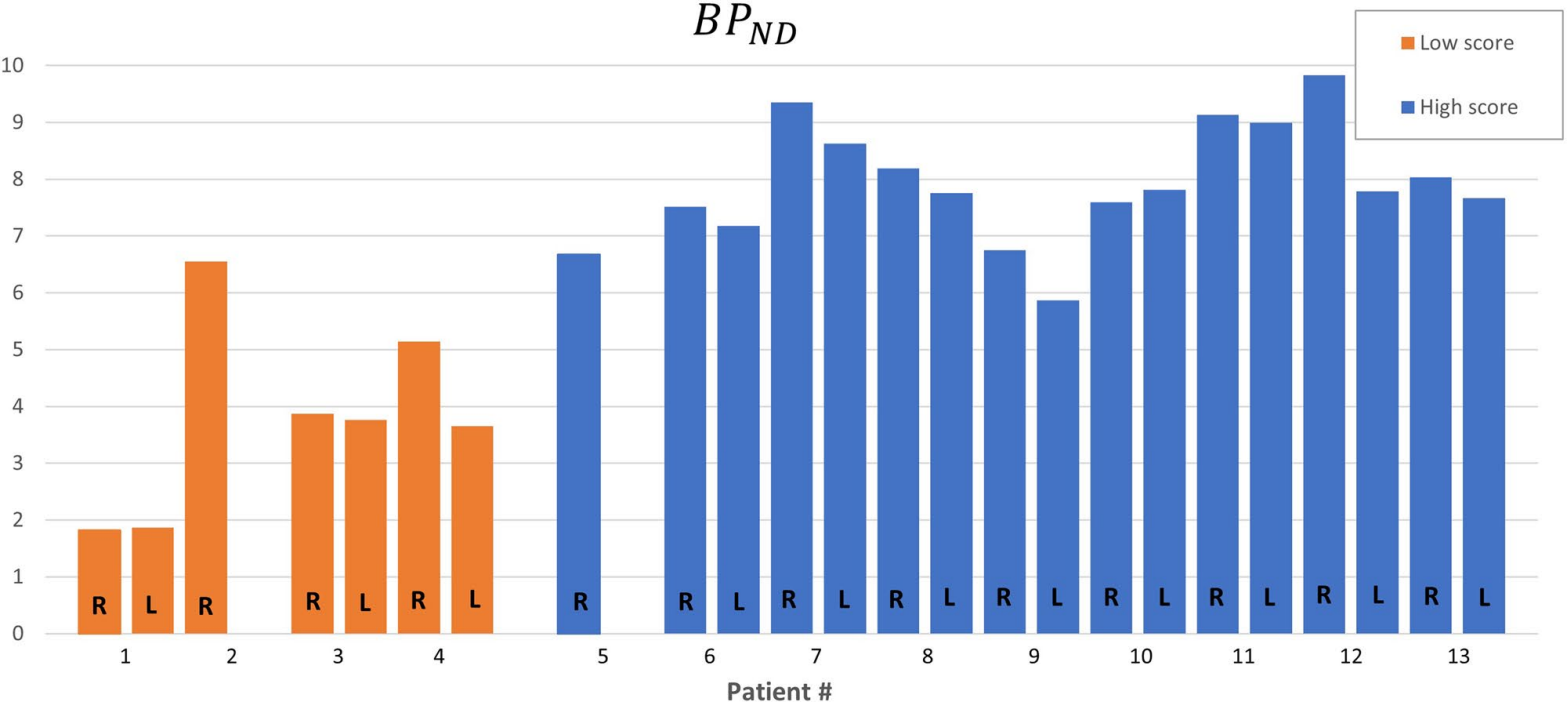
Non-displaceable binding potential (BP_ND_) for each patient’s striata. Each patient is illustrated with a left column representing values for the left striata (N = 11) and a right column representing data for the right striata (N = 13). Patients #1–4 were categorized as low score, and #5–13 were categorized as high score. Right side of #2 and #5 is excluded.

Figure 4 shows the *BP*_ND_ values of each striatum among the patients. LS patients had more putamen-caudate nucleus asymmetry of *BP*_ND_ estimates than HS patients, as shown in Table 3.

**Figure 5:**
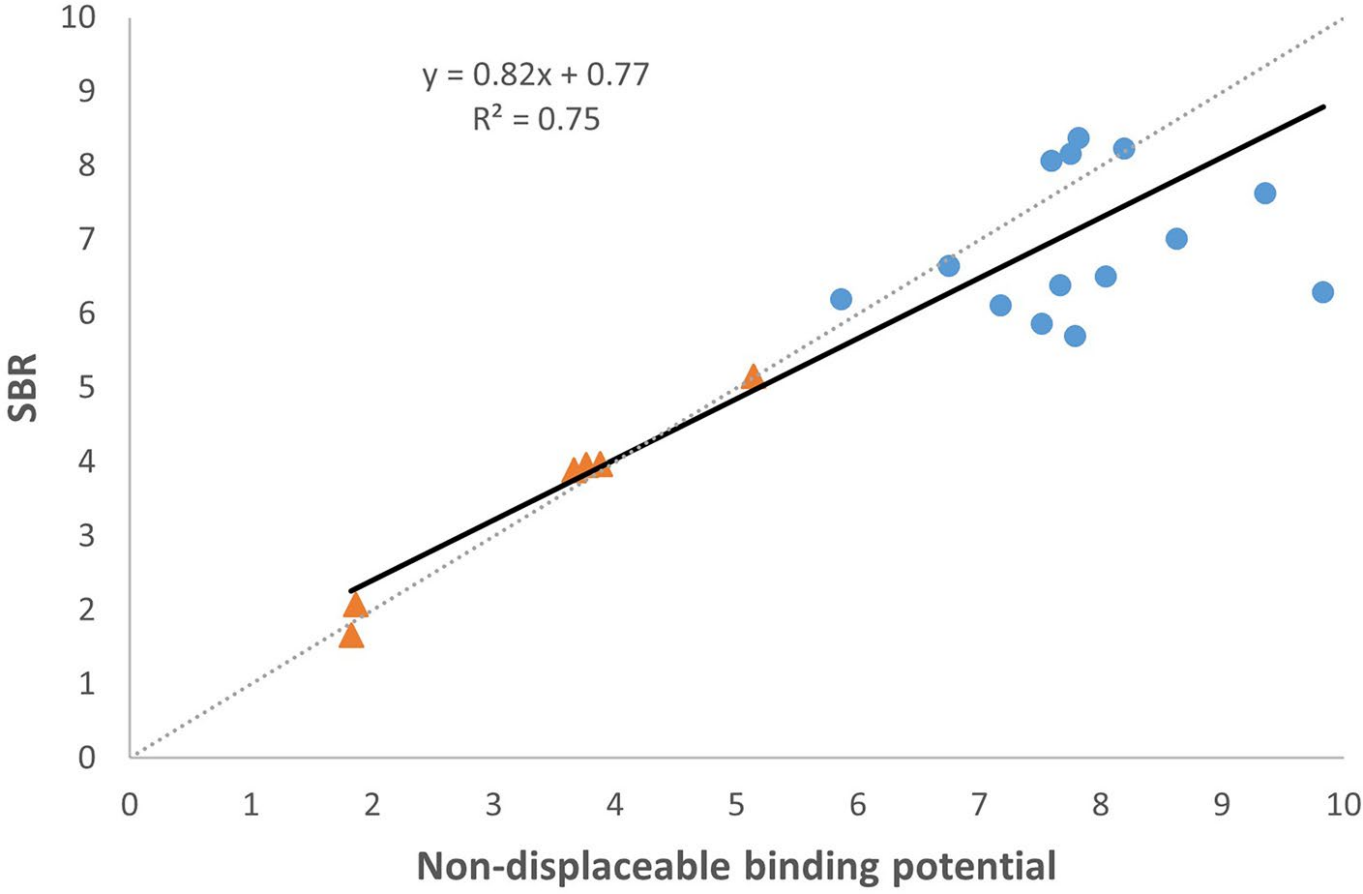
Scatterplot for correlation between measured non-displaceable binding potential (BP_ND_) and calculated specific binding ratio (SBR_calc_). Each data mark is the value of either a left or right striatum from one of the 13 patients with patients #2 and #5 excluded. Orange triangles indicate low-score patients, and blue circles indicate high-score patients.

We compared estimates of SBR_calc_ with estimates of *BP*_ND_ for both left and right striatum, as shown in Fig. 5. The linear correlation had a coefficient of determination (*R*^2^) of 0.75.

We show the *R*_1_ values of all patients in Table 2. Patient #5 with the highest values of *R*_1_ for both left and right striatum had vascular comorbidity in the form of lacunar infarcts. Patient #3 with vascular comorbidity had values close to 1, but with asymmetry of left and right striatum as listed in Table 2. The variability was high among patients for values of *k*_2_, with no clear difference between LS and HS.

### Correlation with clinical presentation

Compared to tremor lateralization, three of the LS group of patients (30% of the total) had lower mean activity concentration in contralateral striatum, while six patients of the HS group (60% of the total) had slightly lower mean activity concentration in ipsilateral hemisphere as listed in Table 2. An LS patient with lateralized tremor had equal activities in left and right striatum, with similar results for SBR_calc_. For *BP*_ND_ and *R*_1_, results varied (Table 2).

## Discussion

The main objective was an examination of the feasibility of functional dynamic brain SPECT (fSPECT) with the three-headed multi-pinhole gamma camera. We created time-activity curves with quantitative uptake values and corrected for PVE. We further showed the feasibility of examining patients in terms of quantitative pharmacokinetic parameters.

We applied standardized volumes of striatum to all patients. The PVE correction by means of Eq. (1) affected all recordings. In the study, we did find correlation between the 3 h p.i. visual assessment by Kahraman et al.^10^ and the striatal mean activity uptake values, SBR_calc_, and the *BP*_ND_ values obtained by SRTM, strongest of which appeared to be the uptake value. In addition, the findings indicate that uptake values distinguished LS and HS patients as early as 2 h p.i. We found reasonable correlation between the dynamic parameter *BP*_ND_ and the SBR_calc_ value. The resulting estimates of the parameters *R*_1_ and *k*_2_ varied among patients but without apparent correlation to the visual assessment.

A correlation between tremor-dominant hemisphere and contralateral declines of both uptake values and SBR_calc_ estimates appeared to exist among LS patients, but none of the kinetic parameters confirmed the correlation.

We examined the dynamic uptakes in both occipital cortex and cerebellum, the latter higher initially, but otherwise similar. As usual with DaT SPECT, we used the occipital cortex as reference. Indeed, in a recent PET study, the occipital cortex emerged as better suited as reference compared to cerebellum, because of slightly higher temporal stability and lower bias^17^.

The imaging protocol outlined previously was chosen as a compromise between image quality and acquisition speed. We recognize that the frame length may remove pharmacokinetic information, however we did not want to sacrifice image quality any further. We did change the imaging protocol halfway through the study, in order to raise the visual presentation of the scan. While the visual presentations did improve slightly, we saw no clear differences among any of the uptake values in striatum, depending on the imaging duration.

Conventional imaging of PD is limited to a simple visual interpretation of tracer uptake at a certain time, often aided by the ratio between activities in striatum and a reference region. The study of quantitative dynamic pharmacokinetic parameters extracted from brains of patients with PD is likely to lead to more nuanced diagnostic tools that improve understanding and differentiation. The binding potential is an index of the numbers of receptors present in a given region and their availability, as a value related to the product of receptor density and affinity of the ligand. Thus, if PD is afflicted by loss of neurons or loss of receptor affinity, the estimate of *BP*_*ND*_ is affected as well. *R*_*1*_ describes relative perfusion and could thus enlight perfusion differences.

In a prior study, Mazère et al. completed dynamic fSPECT imaging with ^123^I-iodobenzovesamicol, a vesicular acetylcholine transporter, in healthy volunteers and patients with Lewy body dementia^18^. The group also managed to create dynamic SPECT image series and to determine *BP*_ND_. In the present study, however, we used half as much injected radioactivity, and the imaging lasted less than half as long. To our knowledge, there are no previous examples of dynamic dopaminergic brain SPECT imaging with pharmacokinetic modelling in the literature. Nonetheless, we can draw parallels to PET imaging. PET studies with the FE-PE2I DaT tracer show significantly lower values of *BP*_ND_ in PD patients compared to healthy control subjects^19,20^. Similarly, we found that values of *BP*_ND_ tended to be lower in patients with lower visual inspection scores. In the present study, we noted an underestimation of activity in striatum by 30% without correction for PVE. A PET study with both ^11^C-FLB457 and ^18^F-fallypride tracers revealed increases of estimates of *BP*_ND_ of 50% by correction for PVE^21^. Likewise, in a prior study of ^123^I-labeled tracer in a brain phantom, the authors reported underestimation of activity in striatum of close to 50%, and further underestimation of *BP* without correction for PVE^22^. The present evidence shows that dynamic brain scans are feasible by fSPECT, thereby increasing the possibilities of performing dynamic brain scans independently of PET-CT.

While a dynamic PET brain scan requires only 60 min of acquisition^5^, the dynamic fSPECT by contemporary protocols requires longer durations, and parkinsonian patients may have difficulty resting on the back for longer periods, also because the patients often have stiff necks and tense shoulders. We did experience several of the patients colliding with the collimators due to tense and raised shoulders, and the movements of the patients’ increased the uncertainty of measurements, also of the effect of small movements on the uptake values. Of the two patients for whom the method and SRTM provided unrealistic and imprecise values of *BP*_ND_, at least one patient had vascular pathology in striatum. Therefore, we need more dynamic studies in case of vascular comorbidity.

Increasing age provides lower DaT availability with as much as 11% decline per decade^23,24^. While both gender and BMI influence DaT availability^24,25^, we now need further studies of these factors to evaluate the effects on dynamic uptake. The correlations between visual assessment and uptake values and *BP*_ND_ warrant consideration of shorter imaging durations, but further studies are needed to assess the imaging durations affecting these parameter estimates, and to determine the imaging durations that limit the determination of values of *BP*_ND_. Previously we thought that estimates of *R*_1_ reflect perfusion or extraction effects^16^, but we were unable here to detect a correlation that may be evident in larger studies.

A neurologist referred all the participants in the study, all as suspected of having PD, although none displayed clear Parkinson-like symptoms that would allow diagnosis on the basis of clinical presentation alone.

### Strengths and weaknesses

The present study is a feasibility study that included only a small number of participants, with no normal control subjects. Nonetheless, as far as we know, the study is the first to confirm the feasibility of dynamic fSPECT.

We gathered no follow-up data to determine whether or not PD was actually diagnosed in any of the participants. We did not collect the participants’ medical journals, and many of the participants were referred by private neurology practitioner that ruled out clinical correlation such as Hoehn-Yahr staging. We obtained no height or weight of the participants, and we had no estimates of blood distribution volume that may have had an impact on the availability of the tracer.

## Conclusion

We tested the claim of using a three-headed SPECT device to obtain fSPECT images with multi-pinhole collimators. The findings show that dynamic brain SPECT with [^123^I]FP-CIT is feasible and that it is possible to obtain values of quantitative uptake and pharmacokinetic parameters from the images. The findings show the feasibility of examining patients suspected of PD in new ways by means of fSPECT. Further studies are warranted to examine the optimal future protocol of fSPECT and to examine the diagnostic value of dynamic parameters.

## Data Availability

The datasets generated during and/or analysed during the current study are available from the corresponding author on reasonable request.

## References

[1] Collaborators, G. B. D. P. s. D. Global, regional, and national burden of Parkinson's disease, 1990–2016: A systematic analysis for the Global Burden of Disease Study 2016. *Lancet Neurol.* **17**, 939–953 (2018). 10.1016/S1474-4422(18)30295-3 10.1016/S1474-4422(18)30295-3 PMC6191528 30287051

[2] Group, G. B. D. N. D. C. Global, regional, and national burden of neurological disorders during 1990-2015: A systematic analysis for the Global Burden of Disease Study 2015. *Lancet Neurol.* **16**, 877-897 (2017). 10.1016/S1474-4422(17)30299-5 10.1016/S1474-4422(17)30299-5 PMC5641502 28931491

[3] Bloem BR, Okun MS, Klein C (2021). Parkinson's disease. Lancet.

[4] Balestrino R, Schapira AHV (2020). Parkinson disease. Eur. J. Neurol..

[5] Morbelli S (2020). EANM practice guideline/SNMMI procedure standard for dopaminergic imaging in Parkinsonian syndromes 1.0. Eur. J. Nucl. Med. Mol. Imaging.

[6] Mathies F (2022). Multiple-pinhole collimators improve intra- and between-rater agreement and the certainty of the visual interpretation in dopamine transporter SPECT. EJNMMI Res..

[7] Thilsing-Hansen K, Simonsen J, Hvidsten S (2021). Performance evaluation of a clinical multi-pinhole brain SPECT system: Comparison with a conventional SPECT system. J. Nucl. Med..

[8] Tecklenburg K (2020). Performance evaluation of a novel multi-pinhole collimator for dopamine transporter SPECT. Phys. Med. Biol..

[9] Nakabayashi S (2018). Denoising projection data with a robust adaptive bilateral filter in low-count SPECT. Int. J. Med. Phys. Clin. Eng. Radiat. Oncol..

[10] Kahraman D, Eggers C, Schicha H, Timmermann L, Schmidt M (2012). Visual assessment of dopaminergic degeneration pattern in 123I-FP-CIT SPECT differentiates patients with atypical parkinsonian syndromes and idiopathic Parkinson's disease. J. Neurol..

[11] Andersen HG (2020). Striatal volume increase after six weeks of selective dopamine D2/3 receptor blockade in first-episode, antipsychotic-naive schizophrenia patients. Front. Neurosci..

[12] Lee SH (2011). Regional volume analysis of the Parkinson disease brain in early disease stage: Gray matter, white matter, striatum, and thalamus. AJNR Am. J. Neuroradiol..

[13] Tossici-Bolt L, Hoffmann SMA, Kemp PM, Mehta RL, Fleming JS (2006). Quantification of [123I]FP-CIT SPECT brain images: An accurate technique for measurement of the specific binding ratio. Eur. J. Nucl. Med. Mol. Imaging.

[14] Innis RB (2007). Consensus nomenclature for in vivo imaging of reversibly binding radioligands. J. Cereb. Blood Flow Metab..

[15] Lammertsma AA, Hume SP (1996). Simplified reference tissue model for PET receptor studies. Neuroimage.

[16] Buchert R, Thiele F (2008). The simplified reference tissue model for SPECT/PET brain receptor studies. Interpretation of its parameters. Nuklearmedizin.

[17] Delva A (2020). Quantification and discriminative power of (18)F-FE-PE2I PET in patients with Parkinson's disease. Eur. J. Nucl. Med. Mol. Imaging.

[18] Mazere J, Lamare F, Allard M, Fernandez P, Mayo W (2017). 123I-Iodobenzovesamicol SPECT imaging of cholinergic systems in dementia with lewy bodies. J. Nucl. Med..

[19] Jakobson Mo S (2018). Dopamine transporter imaging with [(18)F]FE-PE2I PET and [(123)I]FP-CIT SPECT-a clinical comparison. EJNMMI Res..

[20] Fazio P (2015). Quantitative analysis of (1)(8)F-(E)-N-(3-Iodoprop-2-Enyl)-2beta-Carbofluoroethoxy-3beta-(4'-Methyl-Phenyl ) Nortropane binding to the dopamine transporter in Parkinson disease. J. Nucl. Med..

[21] Smith CT (2019). Partial-volume correction increases estimated dopamine D2-like receptor binding potential and reduces adult age differences. J. Cereb. Blood Flow Metab..

[22] Soret M, Koulibaly PM, Darcourt J, Hapdey S, Buvat I (2003). Quantitative accuracy of dopaminergic neurotransmission imaging with (123)I SPECT. J. Nucl. Med..

[23] Pirker W (2000). Imaging serotonin and dopamine transporters with 123I-beta-CIT SPECT: Binding kinetics and effects of normal aging. J. Nucl. Med..

[24] Varrone A (2013). European multicentre database of healthy controls for [123I]FP-CIT SPECT (ENC-DAT): Age-related effects, gender differences and evaluation of different methods of analysis. Eur. J. Nucl. Med. Mol. Imaging.

[25] Lee JJ (2016). Association of body mass index and the depletion of nigrostriatal dopamine in Parkinson's disease. Neurobiol. Aging.

